# Enhanced Anticancer Activity of Nedaplatin Loaded onto Copper Nanoparticles Synthesized Using Red Algae

**DOI:** 10.3390/pharmaceutics14020418

**Published:** 2022-02-15

**Authors:** Nada Mostafa Aboeita, Sherif Ashraf Fahmy, Mayyada M. H. El-Sayed, Hassan Mohamed El-Said Azzazy, Tamer Shoeib

**Affiliations:** 1 Department of Chemistry, School of Sciences & Engineering, The American University in Cairo, AUC Avenue, P.O. Box 74, New Cairo 11835, Egypt; nada.aboeita@aucegypt.edu (N.M.A.); sheriffahmy@aucegypt.edu (S.A.F.); 2 School of Life and Medical Sciences, University of Hertfordshire Hosted by Global Academic Foundation, R5 New Garden City, New Administrative Capital, AL109AB, Cairo 11835, Egypt

**Keywords:** nedaplatin, CuO nanoparticles, cancer cell lines, green synthesis, algal extract, ultrasound-assisted extraction

## Abstract

Marine algae are a rich source of biologically active compounds that can be utilized in various food and pharmaceutical applications. In this study, ultrasound-assisted extraction (UAE) was optimized to maximize yield and total carbohydrate content extracted from the red algae, *Pterocladia capillacea*. The extract was shown to possess potent antioxidant activity of up to ~70%, and was successfully used as a reducing and capping agent in the green synthesis of copper nanoparticles, which were characterized by UV-spectroscopy, Fourier transform infrared (FTIR) spectroscopy, X-ray diffraction (XRD), transmission electron microscopy (TEM), and dynamic light scattering (DLS). Primarily, CuO nanoparticles with an average size of 62 nm were produced. FTIR spectra for the extract and algal-mediated CuO nanoparticles showed characteristic polysaccharide peaks. The synthesized CuO nanoparticles were subsequently loaded with nedaplatin where UV data suggested a complex formation. Nedaplatin release profiles showed a sustained release that reached a maximum at 120 h. The formulation was shown to have greater cytotoxicity relative to nedaplatin on hepatocellular carcinoma, breast cancer and ovarian cancer cell lines with IC_50_ values of 0.40 ± 0.08, 1.50 ± 0.12, and 0.70 ± 0.09 µg/mL, respectively. Loading nedaplatin onto CuO nanoparticles synthesized using red algae extract, greatly enhances its anticancer effect.

## 1. Introduction

Nedaplatin (ND), a second-generation platinum-based anticancer drug, is developed from cisplatin and clinically approved in Japan [1,2]. ND was synthesized to enhance the water solubility of cisplatin and to overcome some of its adverse effects, including nephrotoxicity and gastric toxicity while maintaining a similar pharmacokinetic profile. Additionally, ND, which exerts its cytotoxic activity through the interaction of its activated species forming Pt-DNA adducts, has shown remarkable anticancer activity against cervical, small and non-small cell lung, head, neck, breast, ovarian and testicular cancers [3,4,5]. However, ND resistance and its substantial adverse effects present a significant hindrance to its global acceptance.

Considerable efforts have been exerted to develop delivery systems for chemotherapeutic agents that can overcome their limitations while simultaneously increasing their bioavailability and selective uptake by the targeted cancer cells [6,7,8,9,10,11,12,13]. Many studies have reported the use of algal-mediated metallic oxide nanoparticles (NPs), including copper and zinc oxides, as safe, eco-friendly, and biodegradable nanocarriers for chemotherapeutic agents with superior physicochemical, optical, and biochemical characteristics [14,15]. In that regard, algal species were used as delivery vehicles for docetaxel, paclitaxel, cisplatin, 5-furouracil, doxorubicin, as well as nucleic acid-based drugs [15].

Marine algal extracts obtained from red algae such as *Palmaria palmate, Chondrus crispus,* and *Mastocarpus stellatus* are typically used in the green synthesis of metallic oxide nanoparticles due to their high polysaccharide content, which reduces the metal cations to zero-valent metals while forming a capping layer that stabilizes the metal atoms and prevents their agglomeration. This biogenic synthesis route provides a benign means of creating metallic oxide nanoparticles whereby the health risks associated with the chemical synthesis of nanoparticles can be mitigated [16].

In this work, the crude extract of the red algae *Pterocladia capillacea* was obtained via an eco-friendly ultrasound-assisted extraction (UAE) method. The extract was then used to biosynthesize CuO NPs, which were characterized by various techniques, including UV-spectrophotometry, Fourier transform infrared (FTIR) spectroscopy, X-ray diffraction (XRD), transmission electron microscopy (TEM), and dynamic light scattering (DLS). The NPs were then complexed with ND and the drug release kinetics were studied on the formed complex. In addition, the anticancer activity of the formed complex was examined on HEP-G2, MCF-7 and SKOV-3 cell lines under different concentrations of ND, and compared to equivalent concentrations of non-complexed ND.

## 2. Materials and Methods

### 2.1. Materials

The marine red algae *Pterocladia capillacea* used in this study were collected from the Mediterranean Sea coast at a depth of about 3 m down, in the Montazah area in Alexandria, Egypt. They were then washed, dried and ground. Nedaplatin, 99% was purchased from Shandong Boyuan Pharmaceutical Co. (Shandong Yuhuang Chemical Co., Heze city, China). Copper sulfate pentahydrate, CuSO_4_·5H_2_O, 99% was obtained from Chem-Lab, NV, Zedelgem, Belgium. All other chemicals were of laboratory grade, phenol (BDH Chemicals, Dubai, UAE), sulfuric acid, 98% (Penta, Prague, Czech Republic), Folin and Ciocalteu’s phenol reagent (Loba Chemie, Mumbai, India), ethanol, 96%, and 2,2′-Diphenyl-1-picrylhydrazyl (DPPH) (Sigma-Aldrich, Taufkirchen, Germany). All reagents were used without further purification. For biological testing, sterility was obtained through filtration via sterile nylon 25 mm diameter, 0.22 µm pore size syringe filters (CHM lab group, Barcelona, Spain).

### 2.2. Ultrasound-Assisted Extraction (UAE)

Algae were washed carefully with tap water followed by deionized water to get rid of any dust and debris, then dried and ground ready for extraction. The UAE was conducted by adding 5 g of algae to 100 mL of distilled water and placing the mixture in an ultrasonic bath (Branson 2510-DTH, Cambridge, MA, USA), adjusted to room temperature to avoid thermal degradation of active constituents. Sonication times of 0.5, 1, 2, and 4 h were employed followed by filtration using a cheesecloth and centrifugation at 5000–6000 rpm for 15 min to get rid of the undissolved matter. The extracts obtained at the various sonication times were subsequently freeze-dried for future use [17].

### 2.3. Analysis of the Algal Extracts

#### 2.3.1. Determination of the Total Carbohydrate Content

The phenol–sulfuric method was adopted, with minor modifications, to determine the total carbohydrate content of the algal extract using glucose as a standard [17]. Samples were prepared for the test by adding 0.01 g of the freeze-dried extract that was obtained under different extraction times (0.5, 1, 2, 4 h) to 10 mL of distilled water, and the resulting solutions were then diluted 10 times with water to obtain the aqueous algal extract. This was followed by the addition of 1 mL of phenol solution (5% *v*/*v*) and 5 mL of concentrated sulfuric acid to the sample, which was left in a water bath for 30 min at room temperature. The colored complex was then measured spectrophotometrically at 480 nm [17].

#### 2.3.2. Determination of the Total Phenolic Content

The Singleton and Rossi method was used to determine the total phenolic content (TPC) while using gallic acid (GA) as a standard [18]. The stock solution of gallic acid was prepared by dissolving 0.1 g of gallic acid in 100 mL of ethanol, and different dilutions were prepared. The aqueous algal extract samples were prepared as explained in the previous section. Then, 1 mL of each aqueous extract was added to 5 mL of the Folin–Ciocalteu (1:10) reagent and 4 mL of anhydrous Na_2_CO_3_. The prepared samples were shaken very well and left in the dark for 1 h at room temperature. The reference and the standard samples were similarly prepared and the absorbance was measured at 765 nm [18].

#### 2.3.3. Determination of the DPPH Free Radical Scavenging Activity

The antioxidant activity of the red algal extract of *Pterocladia capillacea* was determined by measuring its free radical scavenging activity using DPPH antioxidant assay, according to Blois with minor modifications [19]. Briefly, 2 mL of a 0.1 M solution of DPPH were added to 2 mL of a freshly prepared aqueous algal extract. Again, the aqueous extracts were prepared as previously mentioned, however at different concentrations of 250, 100, 50 and 25 mg/mL. The samples were shaken then left to settle in the dark for 1 h at room temperature. Upon adding the red algal extract to the DPPH reagent, the color changed from violet to yellow with varying intensities depending on the employed concentration. The absorbance was finally measured at 517 nm using methanol as a blank. The free radical scavenging activity was measured according to the following equation.

(1) $$Scavenging\;rate\;\left(\%\right)=\left[\frac{A_{0}-A_{1}}{A_{0}}\right]\times 100$$

where, *A*_0_ is the absorbance of DPPH and *A*_1_ is the absorbance of DPPH with the sample.

### 2.4. Synthesis of Copper Nanoparticles

A total of 0.02 g of the freeze-dried extract was added to 100 mL of 3 mM copper sulfate solution. The mixture was kept on a magnetic stirrer for 3 h at 70 °C [19]. The solution was then centrifuged at 5000–6000 rpm for 15 min. The residue was discarded, leaving the supernatant, then the solution was poured into dialysis bags and was left overnight. The formed nanoparticles were collected and centrifuged at 15,000 rpm for 5 min (POLYTRON, PT 10-35GT) to get the desired nanoparticle size [20]. The formation of the nanoparticles was confirmed by UV-VIS spectrophotometry (Cary 500 Scan) to detect the peak corresponding to copper nanoparticles.

### 2.5. Characterization of Copper Nanoparticles

To examine the crystallinity and the composition of the synthesized nanoparticles, their dry powders were analyzed by a Bruker D8 Discover X-ray diffractometer that uses CuO K alpha radiation (wavelength = 1.54 Å). FTIR was used to determine the functional groups present in the algal extracts and the prepared copper nanoparticles. The samples were mixed with potassium bromide and pressed into 1 mm pellets and were subsequently analyzed by a TGA/FTIR Nicolet 380 spectrometer in the wavelength range of 500 to 4000 cm^−1^.

A transmission electron microscope (JEOL-JEM-2100) was used to examine the morphology of the formed nanoparticles. The samples were suspended in distilled water and were well dispersed in an ultrasonic bath for 15 min. A few drops of the suspension were placed onto previously prepared grids covered by a thin film of evaporated carbon, then examined under the microscope. The zeta-size and zeta-potential of the prepared nanoparticles, diluted 1:10 (*v*/*v*) using ultrapure water, were measured in triplicate using the dynamic light scattering technique in a Zeta-sizer Nano-ZS90 (Malvern Instruments Co., Worcestershire, UK) at 25 °C [21].

### 2.6. Nedaplatin Loading onto the Nanoparticles

A stock solution of ~1 mM ND was prepared by adding 3 mg of ND to 10 mL of distilled water. From the stock solution, a secondary stock of 0.2 mM of ND was prepared, which was, in turn, used to prepare nine solutions of different ND concentrations in the range of 0.01–0.09 mM [1,22,23,24]. The absorbance of these solutions, corrected for the absorbance of the 0.2 mM of the CuO NPs solution was then used to determine the loading of ND onto CuO NPs [1,22,23,24].

#### 2.6.1. In Vitro Drug Release 

The release profile of ND from CuO NPs-ND was determined at 37.5 °C. Volumes of 0.5 mL of the drug formulation were placed into dialysis bags and each bag was then placed in a beaker containing 25 mL of phosphate buffer solution, pH 7.4. The beaker was sealed and placed in a shaking water bath at 37.5 °C for 72 h. Subsequently, 1 mL samples were withdrawn from the beaker at various time intervals and replaced by fresh buffer. The concentration of ND in the samples collected was measured by UV-spectroscopy using calibration curves [1,2,7].

#### 2.6.2. Cell Culture 

Cell lines were obtained from the American Type Culture Collection (ATCC, Manassas, VA, USA). The cell lines used were MCF-7 breast cancer cell line, HEP-G2 hepatocellular carcinoma cell line, SKOV-3 ovarian cancer cell line, and OEC normal oral epithelial cell line. Cells formed monolayers and were maintained in Dulbecco’s Modified Eagle media supplemented with 100 mg/mL streptomycin, 100 units/mL of penicillin, and 10% of heat-inactivated fetal bovine serum in humidified 5% (*v*/*v*) CO_2_ atmosphere at 37 °C. 

#### 2.6.3. In Vitro Cytotoxicity Tests 

The cell viability was assessed by sulforhodamine B (SRB) assay, where aliquots of 100 μL cell suspension (~5 × 10^3^ cells) were placed in 96-well plates and incubated in complete media for 24 h. Cells were treated with another aliquot of 100 μL media containing ND, CuO NPs, or CuO NPs-ND at various concentrations (0.001, 0.01, 0.03, 0.1, 0.3, 1, 3, 10, 30 and 100 µg/mL). After 72 h, cells were fixed by replacing the media with 150 μL of 10% trichloroacetic acid (TCA) incubated at 4 °C for 1 h. The TCA solution was then removed, and the cells were washed 5 times with complete media. Aliquots of 70 μL SRB solution (0.4% w/v) were added and incubated in the dark at room temperature for 10 min. The plates were washed 3 times with 1% acetic acid and allowed to air-dry overnight. About 150 μL of Tris buffer (10 mM) was subsequently added to dissolve the protein-bound SRB stain. The absorbance was measured at 540 nm using a BMG LABTECH^®^-FLUOstar Omega microplate reader (Ortenberg, Germany) [6,7].

### 2.7. Statistical Analysis

All tests were performed in triplicate and data are presented as mean ± SD. Data analysis was conducted using the two-tailed Student’s *t*-test at a confidence level of 95%.

## 3. Results and Discussion

### 3.1. Algal Extraction

The crude extract of the red algae *Pterocladia capillacea* was obtained via UAE. The process was conducted at room temperature to avoid the degradation of the bioactive compounds at high temperature [24,25,26,27,28], and for different time intervals (i.e., 0.5, 1, 2, and 4 h) to determine the optimum time for producing the maximum yield. As shown in Table 1, the percentage yield increases with increases in the extraction time from 0.5 to 2 h. Prolonging the extraction time to 4 h, however, had no significant effect on the yield (*p* > 0.05). Thus, 2 h was chosen as the optimum extraction time. In previous reports, UAE was favored over the conventional method since it reduces the time of extraction and eliminates the laborious, time-consuming dialysis step. It also facilitates extraction at room temperature, which is particularly important for extracting thermolabile substances [26]. Our previous work shows that the conventional crude extraction of *Pterocladia capillacea* requires 48 h at room temperature [17], hence ultrasonication reduced the extraction time by 24 times. Not surprisingly, optimal extraction times also vary depending on the nature of the precursor materials and the extraction conditions. For algae and other marine organisms, various optimal extraction times may be expected based on the differences in the chemical composition of the organisms and the structural composition of their cell walls. In a previous study, we demonstrated that UAE of the crude extract of *Jania rubens* and *Avicennia marina* (mangroves) required 4 and 0.25 h, respectively [29].

The extraction time is reported to be a main factor in the effective extraction of carbohydrates from algae [28,30]. The carbohydrate content of the extracts obtained here at the different ultrasound extraction time intervals employed are presented in Table 1. An increase in the carbohydrate content of the extract was observed with an increase in the extraction time, reaching its maximum value of 59.3% ± 0.2 at 2 h, after which it marginally declines at the 4 h time point. Therefore, it can be deduced that the 2 h extraction time produces a carbohydrate content that is about 2.5 times higher than that obtained after 0.5 h. The increase in carbohydrate content with increasing extraction time could be due to the breakdown of the algal cell walls causing the release of their carbohydrate contents [28]. This finding is supported by a previous study that reported that increasing the ultrasound extraction time resulted in a significant increase in the carbohydrate content of the *Chlorella* species extracts [28]. Based on the extract yield and carbohydrate content results, both shown in Table 1, the 2 h extraction time was used in this work.

In addition to the carbohydrate content, the TPC was also measured as presented in Table 1. The phenolic content was previously reported to be directly correlated to the antioxidant activity of an extract, since the phenolic groups can scavenge free radicals and act as antioxidants. The mechanism of scavenging can take place either through the transfer of hydrogen from the phenolic groups to the free radicals, or single electron transfer whereby the electron is transferred from the phenolic ring to the free radical converting it into a stable anion [31,32]. The extraction of phenolic compounds from algae was shown to be a function of extraction time and ultrasound extraction power [11]. Table 1 shows a decrease in the total phenolic contents of the extracts obtained with increasing extraction time. This is most likely attributed to the partial degradation of the bioactive constituents in the algal extract due to the ultrasonic waves, as discussed in recent reports [28].

Based on the results compiled in Table 1, the 2 h extraction time point chosen in this work is a compromise between the 1.3 times reduction in the total phenolic compound as compared with a 0.5 h extraction time, and the extraction yield and carbohydrate content increases by about 1.1 and 2.3 times relative to the 0.5 h extraction time, respectively. 

**Figure 1:**
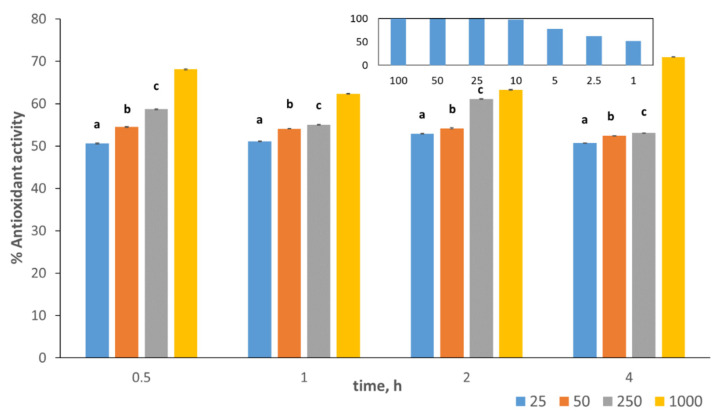
Antioxidant activity (%) of different concentrations of the algal extracts (25, 50, 250, and 1000 μg/mL) obtained after various extraction times, shown in different colors. All experiments were done in triplicate, and values are expressed as mean values ± SD. Groups with the same letters are insignificantly different at *p* = 0.05. The inserted figure shows the % antioxidant activity of different concentrations of ascorbic acid as a reference.

The antioxidant activity of the algal extracts obtained at different extraction times were measured using the DPPH scavenging activity assay employing ascorbic acid as a standard (Figure 1) [32]. For each extraction time, the antioxidant activities were obtained at different extract concentrations of 25, 50, 250, and 1000 μg/mL. A positive correlation between the antioxidant activity and the extract concentration was observed. This finding agrees with recent studies conducted on four algal extracts of Ulva species and for different extracted fractions of polysaccharides obtained from the red algae *Pterocladia capillacea* [31,32].

An inverse relationship between the TPC and extraction time was previously reported for the extraction of fucose from *Laminara digita* and the extraction of polysaccharides from *Rhododendron aganniphum* [33]. However, no clear correlation between antioxidant activity and extraction time was reported in these studies. This might indicate that the phenolic groups are not solely responsible for the observed antioxidant activity, which may also be, in part, attributed to the presence of vitamins such as vitamin C, E, or astaxanthin, a highly potent antioxidant. 

In our work, as shown in Figure 1, at the 2-h extraction time, the average antioxidant activity increased from about 53% to 63% as the extract concentration increased from 25 to 1000 μg/mL. Although a higher antioxidant activity of 71% was obtained at the 4 h extraction time for the same 1000 μg/mL extract concentration, this modest increase in antioxidant activity still does not justify doubling the extraction time.

### 3.2. Algal-Mediated Synthesis of CuO NPs

**Figure 2:**
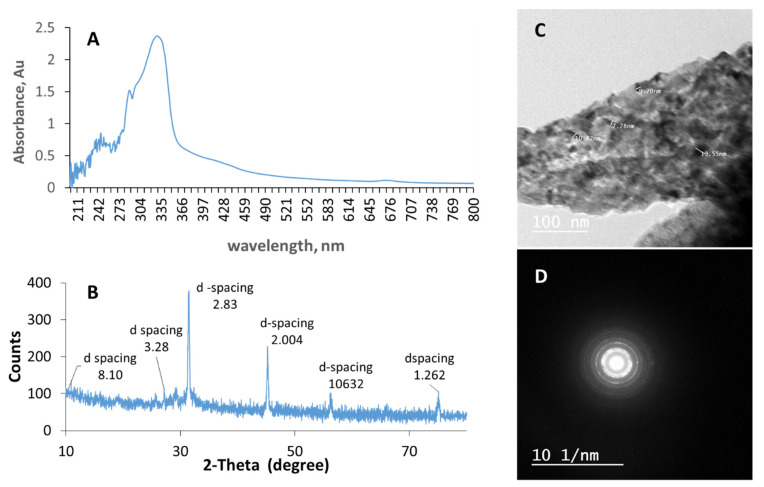
(**A**) UV-vis spectrum, (**B**) XRD pattern, (**C**) TEM image, and (**D**) ring diffraction patterns for the copper nanoparticles.

Figure 2A shows the UV-visible spectrum of the synthesized CuO NPs, where the peak appearing at around 290–330 nm confirms the formation of the nanoparticles. This spectrum showed significant similarity to those attributed to previously synthesized copper nanoparticles from brown algae [34]. The wavelength range is also in good agreement with the 290–320 nm range previously reported for biosynthesized copper nanoparticles and the 250–350 nm range for CuO NPs [35]. 

A representative size distribution curve for the synthesized CuO NPs, as shown in Figure S1, reveals an average particle size of 62 ± 17.7 nm. This is in line with a size range of 40–100 nm previously reported for CuO NPs prepared using the extract of magnolia [36]. Figure S2 shows the zeta potential distribution of the copper nanoparticles, indicating a slightly negative surface charge of −16.8 ± 3.9 mV. Higher charges on the surface of nanoparticles typically increase their stability and reduce potential agglomeration [37]; consequently, this increases their shelf-life [37].

The XRD diffraction pattern for the biosynthesized copper nanoparticles is presented in Figure 2B. The diffraction peaks at 2θ values of 10.9°, 27.1°, 31.5°, 45.2°, 56.3°, and 75.1°, which match the planes of (135), (114), (375), (220), (102), and (105), respectively, could be attributed to the presence of CuO and Cu_2_O. These findings agree with previous reports on the synthesis of CuO NPs using the algal extracts of *Bifurcaria bifurcate* and *Botryococcus braunii* [34]. The high-intensity peak at 2θ of 31.4° most likely indicates the prominent presence of crystalline CuO [34]. In addition, the XRD diffraction patterns of some copper nanopowders obtained from the database of NT Base Co., Ltd. (Seoul, Korea) are reported to contain three peaks at 2θ values of 43.6°, 50.8°, and 74.4°, which is in agreement with the pattern obtained in this study.

The TEM image presented in Figure 2C shows irregular-shaped particles with particle sizes ranging between 8 and 19 nm. This size range is less than that obtained by the zeta-sizer, which measures the hydrodynamic size (i.e., it considers the hydrated sphere layer surrounding the particle). In addition, the presence of bright circular spots observed in the electron ring diffraction pattern of the synthesized CuO NPs presented in Figure 2D may indicate the formation of a polycrystalline material [37]. The TEM size obtained here is in line with previous reports showing 14–24 nm CuO NPs that were not completely spherical when synthesized using small concentrations of the *Rhus coriaria* L extract. However, when synthesized using higher amounts of *Rhus coriaria* L extract, the spherical shape of the nanoparticles was enhanced due to the formation of a capping/coating layer of the extract on the nanoparticles [38].

The FTIR spectra pertaining to the algal extract and the copper nanoparticles are shown in Figure S3. The major peaks, with the exception to those appearing at 2360 and 2339 cm^−1^, which appeared in the algal extract spectrum, were also present in that of the CuO NPs, albeit with lower intensities and slight shifts. This may be attributed to the nature and amount of the proteins, polysaccharides and long chain fatty acids present in the crude extract as compared to those that form a stabilizing layer on the surface of the nanoparticles. These observations are in line with previously reported data [34]. For both the extract and CuO NPs obtained in this work, absorption bands were detected at around 3200–3500 cm^−1^ which are most likely attributed to the stretching vibration of hydroxyl groups (OH) groups [32]. Furthermore, peaks appearing at around 1600–1670 cm^−1^ could correspond to N-H bending vibration in the amide group pertaining to the proteins as previously reported [34]. Our previous work on the polysaccharide extracts of *Pterocladia capillacea* [32] confirmed the presence of proteins in these extracts by chemical analysis using the method of Lowry, Rosenbrough, Farr, and Randall. In addition, the appearance of bands in the range of 1400–1425 cm^−1^ could be mainly attributed to the presence of phenolic groups and uronic acid [32], while those appearing at about 1350–1450 cm^−1^ and 805–900 cm^−1^ may correspond to sulfate and ester sulfate groups, respectively. The IR spectra of the algal extract and CuO NPs also showed peaks at 876.5 and 874.2 cm^−1^, respectively, which could belong to galactose sulfate groups [32]. These findings are supported by our previous work on the polysaccharide extracts of *Pterocladia capillacea*, which confirmed the presence of galactose and uronic acid by HPLC analysis, and the presence of sulfates by chemical analysis using the barium chloride turbidimetric assay [32]. As for the presence of phenolic groups, this was confirmed by the TPC measurements conducted in the current study as discussed earlier.

The main functional groups obtained from the FTIR spectra are summarized in Table 2.

### 3.3. Nedaplatin Loading

The UV absorbance spectra of 0.2 mM ND, 0.2 mM CuO NPs, and several mixtures containing successively increasing concentrations of ND (ranging from 0.01 to 0.09 mM) loaded onto a fixed concentration of CuO NPs of 0.2 mM are shown in Figure S4 [22,23,24]. The spectra of the mixtures show a hyperchromic shift as evident by the increase in absorbance, at the wavelength range between 210–220 nm, with increasing concentration of ND. A similar hyperchromic shift was reported recently for the loading of the same drug onto a different carrier [1]. Previous reports on the loading of platinum-based anticancer drugs onto metal nanoparticles biologically synthesized from algae are limited. Some reports, however, showed that loading of the anticancer drug 5-flouracil onto biologically synthesized chitosan nanoparticles was relatively efficient [39]. The syntheses of chitosan nanoparticles loaded with Paclitaxel [40], as well as curcumin and doxorubicin loaded onto fucoidan nanoparticles for cancer applications, have also been reported with high loading efficiencies [41,42].

In order to examine the correlation between the observed hyperchromic shift and the increasing concentration of nedaplatin obtained in this work, the zero-order spectra of the prepared mixtures were divided by the spectrum of CuO NPs, and the first derivative of the ratio spectra as reported elsewhere were obtained using a scaling factor of 10 and Δλ = 4 nm [22,23,24]. The values of the peak amplitudes, measured at 225 nm, were plotted against the concentration of ND, as shown in Figure S5. The strong linear correlation in this plot suggests the hyperchromic shift observed in the absorbance spectra of the mixtures in Figure S4 was due to the complexation between ND and CuO NPs.

### 3.4. Drug Release Study

**Figure 3:**
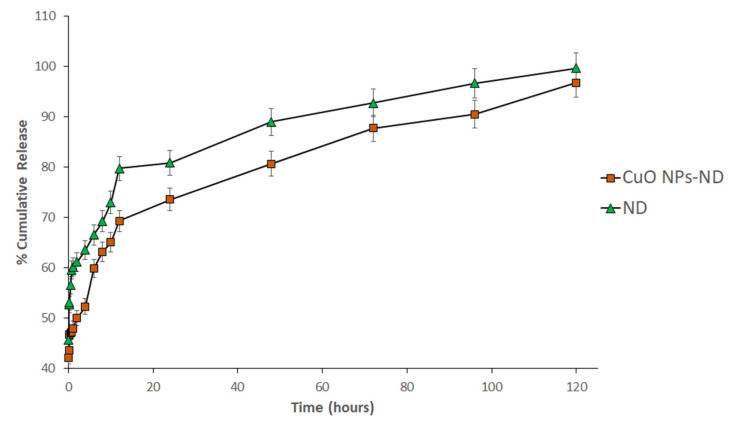
Cumulative release of 0.09 mM nedaplatin formulation from CuO NPs-ND and free ND. Each value shown is the average of triplicate experiments. Error bars represent ± standard deviation.

The release profile of 0.09 mM ND loaded onto the synthesized CuO NPs over 120 h is presented in Figure 3. The profile shows the sustained release of ND from the formulation reaching its maximum release at 120 h. This data is in agreement with previous reports of the sustained release of doxorubicin encapsulated in naturally synthesized fucoidan nanoparticles extracted from brown algae over 5 days [1,43].

### 3.5. Cytotoxicity Studies

The cell viability of hepatocellular carcinoma (HEP-G2), breast adenocarcinoma (MCF-7) and ovarian cancer (SKOV-3) cell lines treated with different concentrations (ranging from 0.001 to 100 µg/mL) of ND, CuO NPs, and the CuO NPs-ND formulation were tested using the sulforhodamine B (SRB) test assay. Some recent reports suggest that the ethanol or ethyl acetate extracts of several algal species display observable cytotoxic activities on several cancer cells [44,45,46]; however, to the best of our knowledge, there are no reports on drugs loaded onto CuO NPs synthesized from algal extracts.

**Figure 4:**
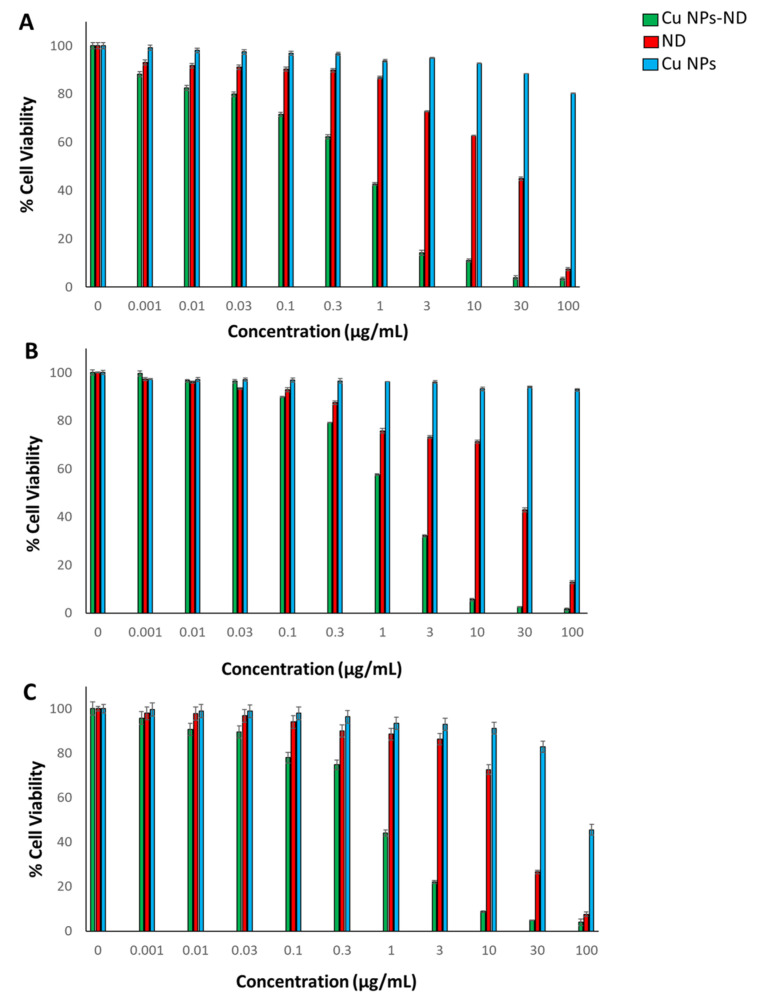
Percent cell viability of (**A**) HEP-G2, (**B**) MCF-7, and (**C**) SKOV-3 cell lines. All the experiments were carried out in triplicate with mean values presented and error bars represent ± standard deviation.

Figure 4A shows the cell viability of HEP-G2 treated with ND, CuO nanoparticles, and the CuO NPs-ND formulation. The treated cell line shows a statistically significant decrease (*p* < 0.05) in cell viability when treated with the CuO NPs-ND formulation with an IC_50_ of 0.4 ± 0.08 µg/mL compared to those treated with equivalent concentrations of ND, which showed an IC_50_ of 14.5 ± 1.73 µg/mL. It is also interesting to note that the CuO NPs alone did not show any significant effect on cell viability. These results indicate that the loading of ND on CuO NPs resulted in a statistically significant decrease in cell viability compared to ND. Figure 4B shows the cell viability of the MCF-7 cell line when treated with ND, CuO NPs, and the CuO NPs-ND formulation. The treatment of the cell line with the CuO NPs-ND formulation resulted in a statistically significant decrease (*p* < 0.05) in the cell viability with an IC_50_ of 1.5 ± 0.12 µg/mL while those treated with equivalent concentrations of ND showed an IC_50_ of 16 ± 0.22 µg/mL. This indicates the degree of potency of the CuO NPs-ND formulation. Figure 4C shows the cell viability of the SKOV-3 cell line ND, CuO NPs, and the CuO NPs-ND formulation. The data presented shows a statistically significant decrease (*p* < 0.05) in the viability of cell lines treated with the CuO NPs-ND formulation relative to that induced by ND, giving an IC_50_ of 0.7 ± 0.09 µg/mL for the formulation and a value of 16 ± 0.18 µg/mL for ND. Similar findings were previously reported on the antiproliferative effect of nanoparticle encapsulated 5-FU exerted on MCF-7 cell lines relative to 5-FU [40]. The data shows that the produced formulation was shown to have significant increased cytotoxic effects on the cancerous cell lines investigated here. Hepatocellular carcinoma cells showed the highest sensitivity to the formulation synthesized here with an IC_50_ of 0.4 ± 0.08 µg/mL compared to IC_50_ values of 0.7 ± 0.09 µg/mL and 1.5 ± 0.12 µg/mL for ovarian cancer and breast adenocarcinoma cell lines, respectively. It is worth mentioning that the CuO NPs-ND formulation is more cytotoxic against multiple cell lines as compared to other reported platinum-based drugs encapsulated in different carriers (Table 3) [47,48,49,50].

## 4. Conclusions

This study presents UAE as an ecofriendly and convenient method of extraction for the red algae *Pterocladia capillacea*. UAE overcomes the potential problems associated with using conventional extraction techniques, such as the use of solvents and elevated temperatures that can damage the bioactive components in algal extracts. The extracts obtained at different extraction time durations using UAE were assessed, showing that extracts after 2 h gave the maximum yield and total carbohydrate content. The extracts were examined for their antioxidant activity using DPPH assay, and it was shown that the antioxidant activity is dependent on the concentration. The extracts were shown to reduce copper ions into copper nanoparticles, which were capped and stabilized, as was confirmed by using different characterization methods. The copper nanoparticles were characterized by UV-spectroscopy, FT-IR spectroscopy, XRD, TEM, and dynamic light scattering. Nanoparticles constituted mainly of CuO with an average hydrodynamic size of 62 ± 17.7 nm were formed. The formed nanoparticles were loaded with nedaplatin. A release study of the loaded nanoparticles showed the sustained release of nedaplatin, reaching a maximum at 120 h. Cytotoxicity assays were performed on different cell lines, including HEP-G2, MCF-7, SKOV-3 and the results showed increased cell death for the loaded nanoparticles relative to nedaplatin. The highest cytotoxicity effect was exhibited on the hepatocellular carcinoma cell line (HEP-G2) with an IC_50_ of 0.4 ± 0.08 µg/mL. This work shows that the biological synthesis of metal nanoparticles from algal extracts may be of great importance in synthesizing other metallic nanoparticles, which may be used in many applications, including the delivery of chemotherapeutics.

## Figures and Tables

**Table 1:** Yield, carbohydrate, and total phenolic content (TPC; expressed as mg gallic acid/g) of the algal extracts at different extraction time points.

Time, h	% Yield	% Carbohydrate	TPC, mgGA/g
0.5	14.2 ± 0.1	24.08 ± 0.03	12.36 ± 0.03
1	15.10 ± 0.03	46.6 ± 0.4	9.72 ± 0.03
2	16.10 ± 0.04	59.3 ± 0.2	9.48 ± 0.04
4	16.08 ± 0.05	55.1 ± 0.1	9.44 ± 0.06

**Table 2:** Main functional groups of the algal extract and copper oxide nanoparticles.

Functional Group	Wavenumber (cm^−1^)	Bond	Extract	CuO NPs
Hydroxyl group	3500–3000	O-H	√	√
Amide group	1670–1600	C=O	√	√
Sulfate	1450–1350	S=O	√	√
Acidic polysaccharide	1120–1000		√	√
Ester sulfate	805–900	C-O-S	√	√
Aromatic ester	1310–1250	C=O	√	X

**Table 3:** Comparison of different Pt-based drugs encapsulated in various carriers against different cancer cell lines.

Platinum-Based Drug	Carrier	Cancer Cells	IC_50_ (µg/mL)	Reference
Cisplatin	Herceptin targeted, diglycolamic acid (DGA) functionalized polyamidoamine (PAMAM) dendrimer	SKOV-3	6.6	[47]
Cisplatin	Solid lipid nanoparticle	MCF-7	6.51	[48]
Oxaliplatin	Carboxylato-pillar [6] arene	HEP-G2	7.6	[49]
Nedaplatin	Cucurbit [7] uril	MCF-7	11.8	[50]
Nedaplatin	CuO NPs	HEP-G2 MCF-7 SKOV-3	0.4 1.5 0.7	This study

## Data Availability

Data sharing is not applicable to this article.

## Supplementary Materials

The following supporting information can be downloaded at: https://www.mdpi.com/article/10.3390/pharmaceutics14020418/s1, Figure S1: Size distribution curve for the biosynthesized copper nanoparticles at 25 °C and neutral pH, Figure S2: Zeta-potential for the biosynthesized copper nanoparticles at 25 °C and neutral pH, Figure S3: FTIR spectra of the algal extract (top panel) and Cu NPs (bottom panel), Figure S4: Absorbance spectra of 0.2 mM nedaplatin, 0.2 copper nanoparticles and several mixtures containing in-creasing concentration (ranging from 0.01–0.09 mM) of nedaplatin and a fixed concentration of 0.2 mM copper nano-particles, Figure S5: Peak amplitudes at 225 nm obtained from the mixture containing increasing concentration of nedaplatin from 0.01 mM to 0.09 mM and a 0.2 mM fixed concentration of copper nanoparticles.
