# Supplementary material for: Enhanced Anticancer Activity of Nedaplatin Loaded onto Copper Nanoparticles Synthesized Using Red Algae
Source: Pharmaceutics. 2022 Feb 15;14(2):418. doi: 10.3390/pharmaceutics14020418 (PMC8877422; doi:10.3390/pharmaceutics14020418)
Supplement: Supplementary file 1 [file pharmaceutics-14-00418-s001.zip › pharmaceutics-1549861-supplementary.pdf]

pharmaceutics

MDPI

Supplementary Materials: Enhanced Anticancer Activity of Nedaplatin Loaded Onto Copper Nanoparticles Synthesized Using Red Algae

Nada Mostafa Aboeita, Sherif Ashraf Fahmy, Mayyada M.H. El-Sayed, Hassan Mohamed El-Said Azzazy, Tamer Shoeib

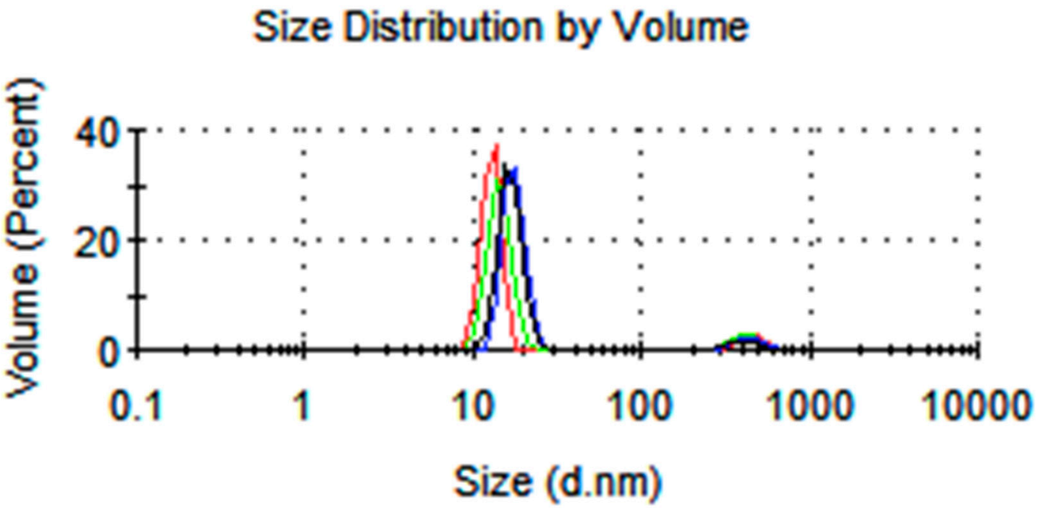

Figure S1. Size distribution curve for the biosynthesized copper nanoparticles at 25 oC and neutral pH.

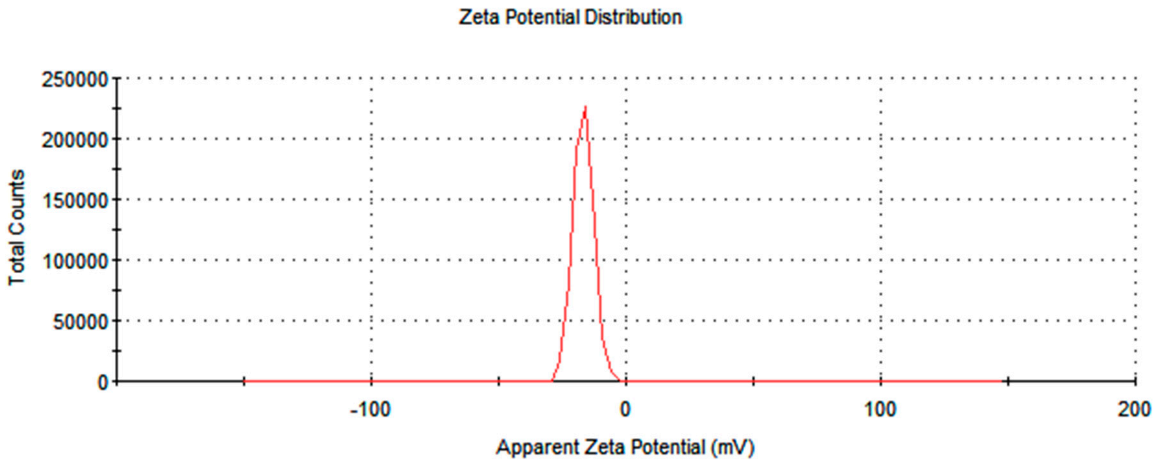

Figure S2. Zeta-potential for the biosynthesized copper nanoparticles at 25 oC and neutral pH.

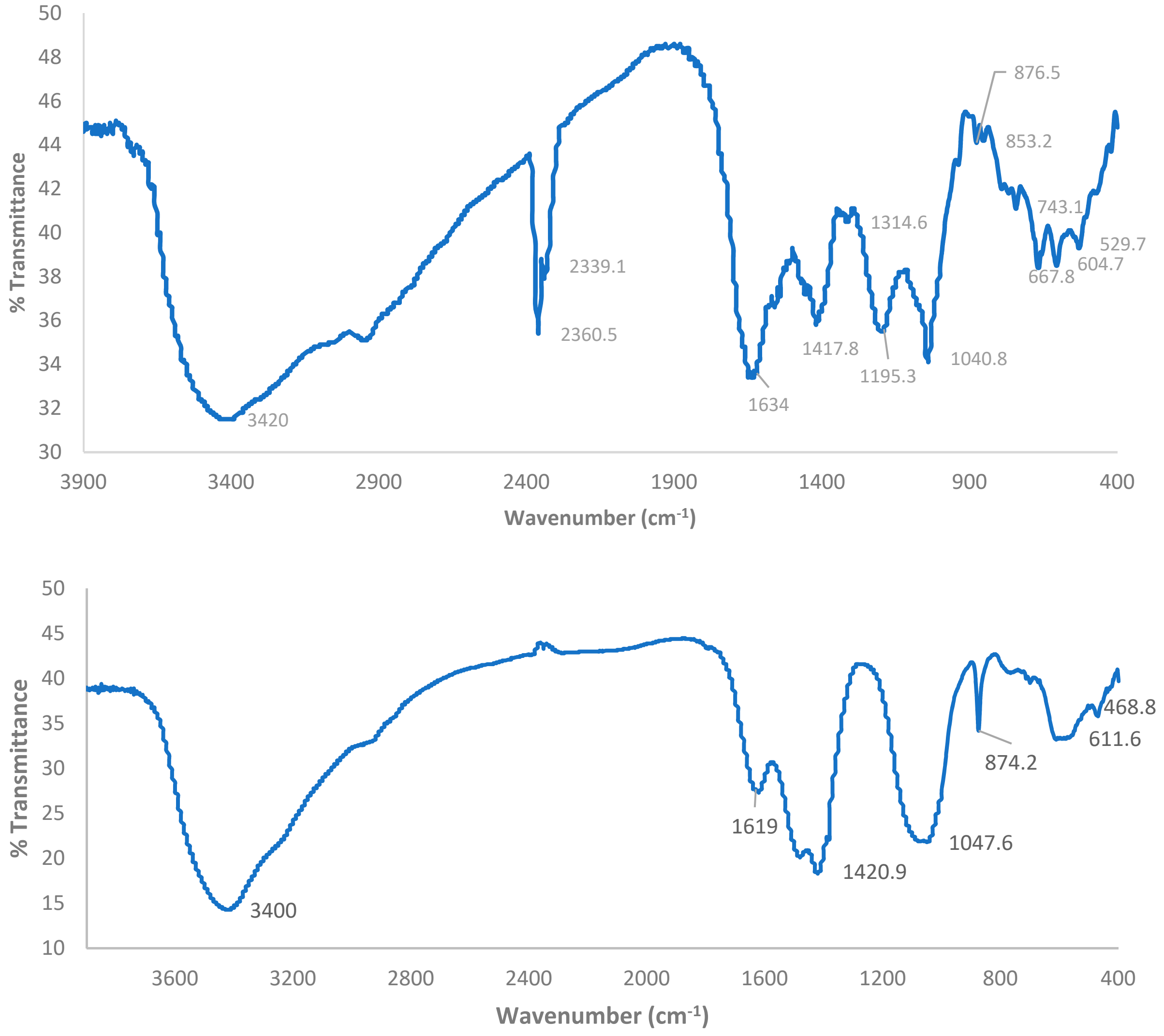

Figure S3. FTIR spectra of the algal extract (top panel) and Cu NPs (bottom panel).

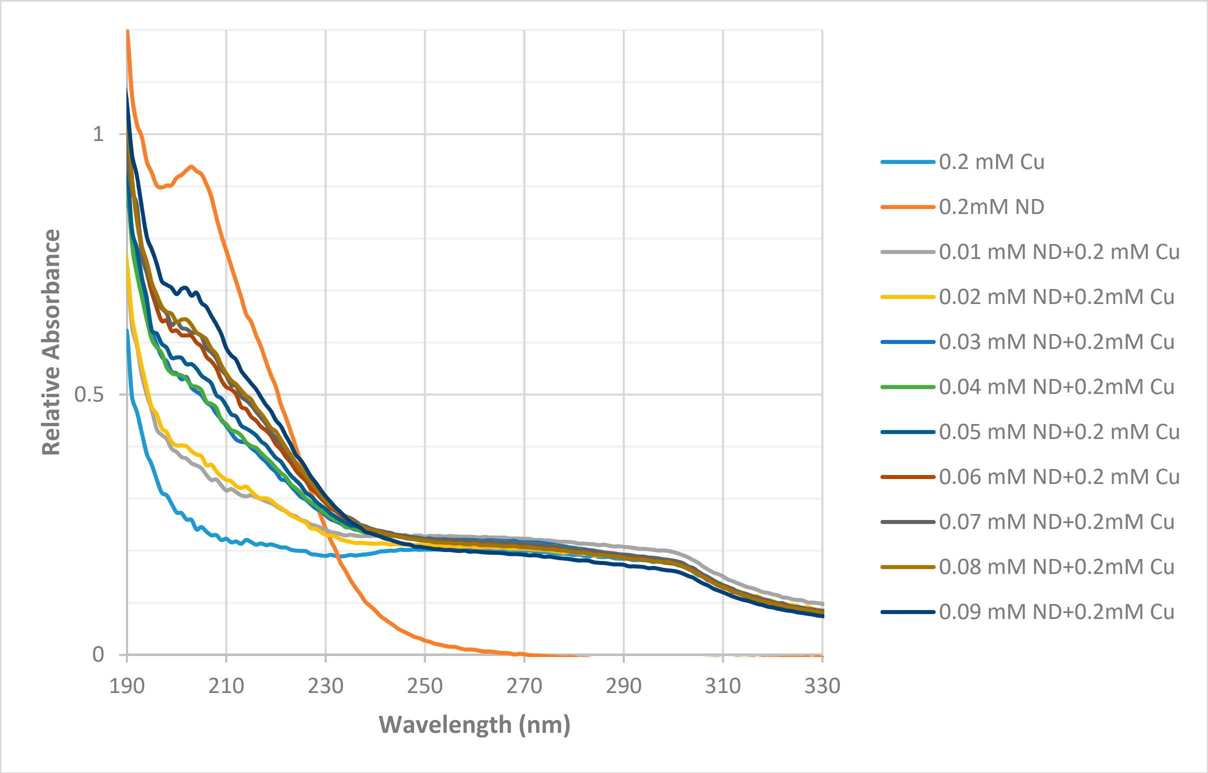

Figure S4. Absorbance spectra of 0.2 mM nedaplatin, 0.2 copper nanoparticles and several mixtures containing increasing concentration (ranging from 0.01-0.09 mM) of nedaplatin and a fixed concentration of 0.2 mM copper nanoparticles.

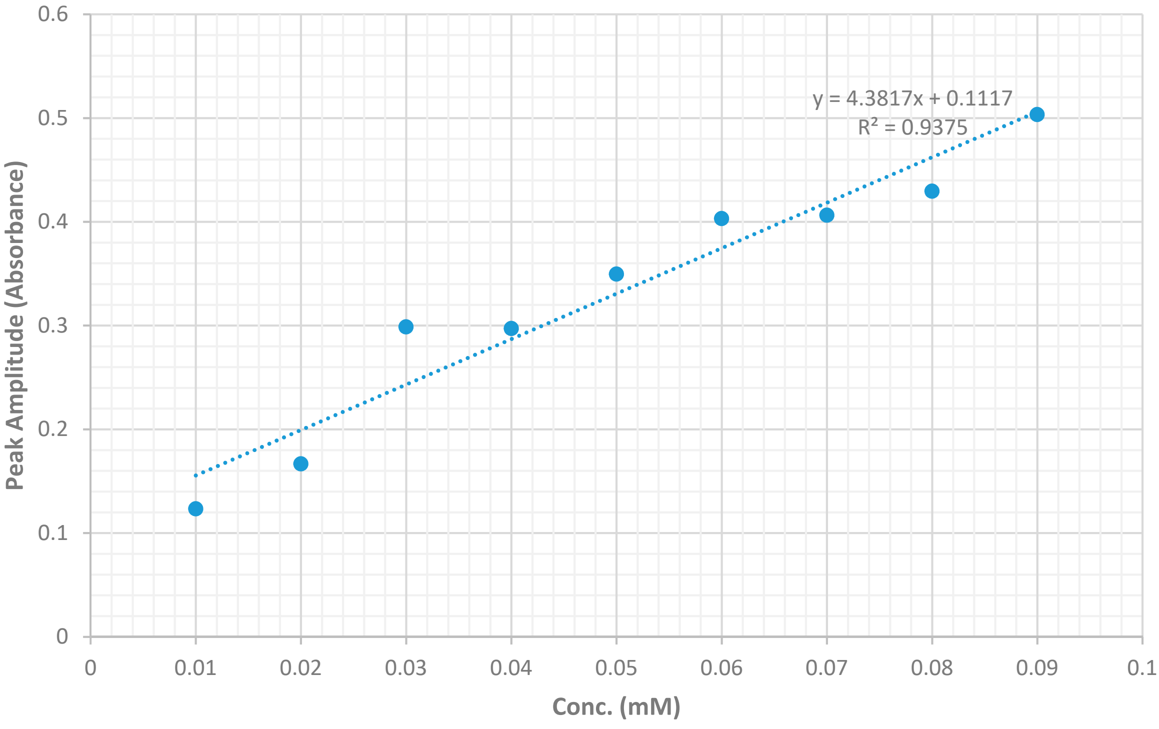

Figure S5. Peak amplitudes at 225 nm obtained from the mixture containing increasing concentration of nedaplatin from 0.01mM to 0.09 mM and a 0.2 mM fixed concentration of copper nanoparticles.
